# *STAG1* Disease, Central Precocious Puberty, and Bone Fragility—A Case Report

**DOI:** 10.3390/diagnostics15091076

**Published:** 2025-04-24

**Authors:** Rebecca-Cristiana Șerban, Andreea-Mădălina Mituț-Velișcu, Andrei Costache, Luminița-Nicoleta Cima, Carmen Niculescu, Aritina Moroșanu, Anca-Lelia Riza, Ioana Streață

**Affiliations:** 1 Laboratory of Human Genomics, University of Medicine and Pharmacy of Craiova, 200638 Craiova, Romania; rebecca.serban@umfcv.ro (R.-C.Ș.); ioana.streata@umfcv.ro (I.S.); 2 Regional Centre of Medical Genetics Dolj, Emergency County Hospital Craiova, 200642 Craiova, Romania; andreea.crgm@gmail.com; 3 Department of Biophysics, University of Medicine and Pharmacy of Craiova, 200638 Craiova, Romania; 4 Department of Endocrinology and Diabetes, Nutrition and Metabolic Diseases, “Elias” Emergency University Hospital, 011461 Bucharest, Romania; luminita.cima@gmail.com; 5 Department of Endocrinology and Diabetes, Nutrition and Metabolic Diseases, Carol Davila University of Medicine and Pharmacy, 050474 Bucharest, Romania; 6 Department of Pediatrics, University of Medicine and Pharmacy of Craiova, 200638 Craiova, Romania; carmen.niculescu@umfcv.ro (C.N.); aritina.morosanu@umfcv.ro (A.M.)

**Keywords:** *STAG1*, cohesinopathy, neurodevelopmental delay, central precocious puberty, bone fragility, case report

## Abstract

**Background**: Previously reported *STAG1* gene-related cohesinopathies describe a range of clinical features, typically including intellectual disability (ID), facial dysmorphisms, and limb anomalies. **Case presentation**: We present the case of an 8-year-old girl with main findings including ID, central precocious puberty (CPP), and bone fragility. Panel genetic testing revealed a pathogenic *STAG1* variant, NM_005862.3:c.2116del p.(Asp706Ilefs*15), which can only partially explain the clinical phenotype. Reports of *STAG1*-related cohesinopathies, including ours, have consistently described developmental and intellectual disabilities. In our case, the etiology of CPP and bone fragility remains unexplained. We discuss the challenges and limitations of current molecular tools in assessing cases with overlapping, apparently unlinked phenotypes, while speculating whether the common occurrence could be explained by *STAG1* instead. **Conclusions**: The clinical spectrum of cohesinopathies is still poorly understood. Complex phenotypes with apparently unrelated clinical features warrant further careful investigation and illustrate the challenges of molecular diagnosis.

## 1. Introduction

Stromal antigen-1, *STAG1*, encodes a component of the cohesin complex, which is crucial for chromosome segregation, DNA repair, and transcriptional regulation [1]. Pathogenic variants in *STAG1* disrupt these processes and are involved in cohesinopathies, rare genetic conditions. They have been increasingly recognized as a cause of neurodevelopmental disorders, typically presenting with intellectual disability (ID), developmental delay, and behavioral abnormalities [2,3]. Craniofacial abnormalities have also been described [4,5,6]. These features overlap clinically with Cornelia de Lange Syndrome (CdLS), for instance, but often present with milder dysmorphic features and predominant neurodevelopmental impairment [4,7,8,9].

However, knowledge on the clinical spectrum of *STAG1*-related disorders is limited. The rarity and clinical variability of *STAG1*-related disorders pose significant challenges to diagnosis and management. The low number of reported cases further underscores the need for comprehensive genotype–phenotype studies in order to better understand the full spectrum of *STAG1*-related disease [6,10].

We present a pediatric case of a rare genetic syndrome with an atypical association of features: clinical findings overlapping with *STAG1* disease-associated phenotype, alongside central precocious puberty (CPP) and bone fragility, neither of which have been previously reported in *STAG1* cohesinopathies.

These conditions may or may not be correlated with each other. Given their intricate underlying mechanisms, it is challenging to distinguish their precise etiology. For instance, CPP, which is characterized by the early onset of puberty caused by premature activation of the hypothalamic–pituitary–gonadal (HPG) axis, can be linked to genetic [11], environmental [12], or central nervous system factors [13]; genetic and metabolic disorders, hormonal imbalances, and chronic disease [14,15] can lead to bone frailty.

Finding efficient diagnostic and management strategies requires a comprehensive understanding of these diverse etiologies, especially in pediatric populations. Starting from the “research/case gap” in this patient, we discuss the challenges and limitations of current molecular tools in assessing cases with apparently unlinked phenotypes, while speculating whether the common occurrence could be explained by *STAG1* instead.

## 2. Case Presentation

### 2.1. Clinical Findings

In 2022, an 8-year-old girl was clinically evaluated at the Regional Centre of Medical Genetics Dolj, Emergency Clinical County Hospital Craiova. The patient was referred to genetics from the Endocrinology Department due to clinical and biological signs of CPP: adrenarche, menarche, leukorrhea, and abnormal hormonal measurements.

**Figure 1:**
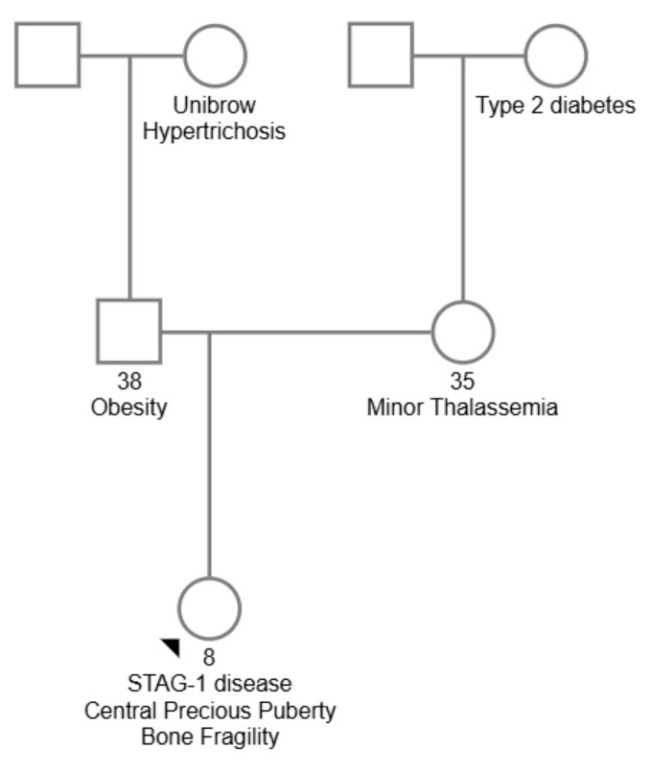
Family pedigree chart for the reported case. 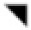 indicates who the proband is.

The proband was a Romanian girl born to healthy, non-consanguineous parents with a negative family history of congenital anomalies, genetic disorders, epilepsy, or ID. Relevant familial traits included maternal minor thalassemia and type 2 diabetes mellitus in the maternal grandmother. On the paternal side, synophrys and hirsutism were noted in the paternal grandmother and great-grandmother, along with a tendency for excessive hair growth. Figure 1 shows the genealogical tree of the family.

She was born by cesarean section following a normal full-term pregnancy, with a birth weight of 3200 g (10th centile) and a length of 50 cm (25th centile). Occipitofrontal circumference was not reported. There were no remarkable events during the perinatal period. In addition, the parents reported frequent respiratory tract infections and anxiety. There was a negative history for head trauma, seizures, encephalitis, or radiation exposure. Her past developmental milestones were within normal limits.

At the time of the initial evaluation in 2022, her height was 138 cm, her weight was 50 kg (50–75th percentile), and her BMI was 26.25 kg/m^2^. The patient had experienced moderate weight gain in the two years leading up to her first presentation.

The patient exhibited moderate facial dysmorphism, including relative microcephaly, a prominent forehead, deep-set eyes, synophrys, a bulbous nose tip, upper lip pilosity, a short neck, a low posterior hairline, and dental caries. Spindle fingers and brachydactyly were also noted. The evaluation of the progression of puberty and sexual maturation on the Tanner scale revealed stage P3-4–pubic hair that was dark, course, and curled and B1-2–breasts that were small mounds; axillary hair was present, as well as apocrine odor, and there was no menarche. Hypertrichosis was present on the legs and paravertebral thorax, and red stretch marks were found on the breasts, arms, and buttocks. A neurological examination revealed anxiety and nocturnal enuresis. The psychiatric assessment noted mild intellectual disability and ADHD. A psychological evaluation indicated Childhood Emotional Disturbance with ID (borderline IQ of 85). She also had a history of multiple fractures from falls from her own standing height, which were treated with plaster casts.

### 2.2. Diagnostic Assessment

**A basal hormonal levels** assessment confirmed CPP based on high levels of luteinizing hormone (LH) and follicle-stimulating hormone (FSH), as well as elevated circulating sex hormones for her age: LH: 5.4 mIU/mL; FSH: 5.7 mIU/mL; estradiol: 164 pmol/L; testosterone: 0.498 nmol/l; DHEAS: 74 µg/dL. Thyroid and adrenocortical functions were normal.

**Imaging**. Ultrasound imaging revealed a hypoechoic area in the right retroareolar region and an endometrial thickness of 4 mm. Hand radiography showed advanced bone age (11.5 years vs. a chronological age of 7 years, 11 months), although this discrepancy was not a consistent finding in later assessments. Brain and hypothalamic-pituitary MRI did not identify any structural alterations but suggested a possible microadenoma, inconclusive due to significant motion artifacts. The MRI was performed at a different center, and the family has not agreed to a second MRI investigation to date.

Electroencephalography (EEG), electrocardiogram, and echocardiogram were unremarkable; similarly, both sight and hearing evaluations were normal.

**Genetic diagnosis.** Genetic testing options were considered, and a step-by-step approach was preferred by the family.

Multiplex Ligation-dependent Probe Amplification (MLPA) for microdeletion syndromes found no deletions/duplications.

Next-generation sequencing (NGS) gene panel testing was subsequently performed. The rare variants (with an overall minor allele frequency below 1%) were evaluated and categorized in accordance with the American College of Medical Genetics recommendations [16]. A gene panel test, including *CYP19A1* and other genes associated with CPP, was initially assessed with a negative result. Subsequent gene panels for disorders of sex development and neurodevelopmental disorders identified a pathogenic variant in *STAG1*: NM_005862.3:c.2116del p.(Asp706Ilefs*15)—a rare frameshift mutation in a highly conserved region of the gene, resulting in an early truncation of the protein [6]; Sanger sequencing performed for the trio (the child and both parents) proved its *de novo* status. The identified variant is associated with Intellectual Disability Disorder, Autosomal Dominant 47.

Since the diagnosis offered only a partial explanation of the clinical presentation, whole-exome sequencing (WES) was performed. WES, using the Ampliseq Exome Panel for Illumina and the AmpliSeq Library PLUS for Illumina (Illumina, San Diego, CA, USA), confirmed the initial finding but did not identify additional genetic variation potentially linked to CPP, bone frailty, or other clinically relevant conditions.

Given that genetic findings do not fully explain the phenotype, periodic clinical follow-up associated with the reinterpretation of these results need to be conducted.

**Diagnostic reasoning**.

The genetic findings partly explain the girl’s phenotype. The identified *STAG1* pathogenic variant may explain several of the features documented in our case.

Many individuals with cohesin complex gene-related syndromes present distinctive facial features, such as hypertelorism, a broad nasal bridge, and a prominent forehead, as well as seizures, growth abnormalities, or endocrinological disorders [3,8,17,18,19]. Previous reports have described language deficits, hypotonia, and ID in individuals with *STAG1* pathogenic variants. In our case, the overlap with clinical features present in Intellectual Disability Disorder, Autosomal Dominant 47 includes variable dysmorphic features and mild ID, but not necessarily all the traits previously described as associated with this pathology; seizures and cerebral anomalies were not found in our patient. Conversely, CPP and bone frailty have not been reported among *STAG1* disease-associated clinical features.

The clinically documented early onset of puberty is diagnosed as CPP based on the laboratory workup. Expectedly, the sex hormone levels seen in our case are comparably higher than the reference levels for the patient’s age [20]. Basal LH levels greater than 0.3 IU/L and a peak LH/FSH ratio ≥ 0.66 confirm the activation of the HPG axis [21] and are used as diagnostic criteria for CPP [22]. The gonadotropin-releasing hormone (GnRH) Stimulation Test, a pivotal test in diagnosing CPP, was not performed. A peak LH level of ≥5 IU/L after GnRH stimulation is indicative of CPP, while a peak LH level of <5 IU/L suggests a prepubertal state [23]. This constitutes a limitation for a definitive diagnosis of CPP. A significantly advanced bone age compared to chronological age is a common finding in CPP and supports the diagnosis [23]. Imaging studies, particularly brain imaging, are important for identifying central lesions that may be causing premature activation of the HPG axis, and thyroid function tests are recommended to rule out other underlying conditions [24]. Pelvic ultrasonography is used to assess the size of the uterus and ovaries, which are typically enlarged in CPP, as observed in our patient as well.

The apparently atypical association among dysmorphic features, ID, CPP, and bone frailty may or may not be in a syndromic context; different factors may contribute to the unexpected, complex phenotype, and, therefore, it is possible that the clinical presentation cannot be explained by a unique etiology.

Interestingly, the patient’s personal and medical history revealed exposure to factors independently linked to the development of CPP, particularly in girls, such as cesarean section and increased body mass index (BMI). Environmental factors, such as diet [25], are also not to be overlooked, although it is not clear how they interfere with the development of CPP. Notably, our patient was slightly overweight at the initial evaluation and continued to gain weight. Nonetheless, a higher BMI, over 30 kg/m2, is typically considered a risk factor; this was not the case at the initial presentation or in the years prior.

### 2.3. Therapeutic Intervention and Follow-Up

Due to the combination of hormonal profile, onset of menarche, and clinical findings, treatment with GnRH analogues (Diphereline–3.75 mg at 26 days) was initiated in September 2021.

In February 2022, at 8 years old, her height was 140.6 cm, her weight was 56 kg, her BMI was 28.3 kg/m^2^, and her arm span was 141 cm. She was at Tanner stage B3, with breast and areola enlargement with no contour differences, and P3, with an increased pubic hair amount that was darkening and starting to curl.

In March 2023, therapy was discontinued due to minimal height gain, persistent bone age advancement (13 years vs. chronological age of 9.5 years), and incomplete hormonal suppression (estradiol 18 pg/mL). At that time, her height was 143.5 cm, weight was 63 kg, and BMI was 30.2 kg/m^2^. She continued to experience recurrent fractures from minor falls and exhibited mild blue sclerae.

In a follow-up visit at 12 years of age, we observed that the patient’s communication, learning, and cognitive skills are delayed. The physical examination noted obesity, noteworthy facial dysmorphism, and the neurological and endocrinological phenotype described above. The progression of puberty was advanced: Tanner stage B4—nipples and areolas are elevated and form an edge toward the breast and the breast has grown slightly larger; P4—pubic hair is denser, with curls and dark hair, along with insulin resistance and a slightly increased total testosterone level.

## 3. Discussion

### 3.1. STAG1 Molecular and Functional Insights–“Typical” Clinical Features

*STAG1* and *STAG2* are stromal antigen proteins with functional specificity in the cohesion complex, which plays a pivotal role in sister chromatid cohesion, DNA repair, and gene expression [1]. They are mutually exclusive subunits, meaning the cohesin complex contains either one or the other, but not both. The cohesin complex influences gene expression by organizing chromatin structure and facilitating long-range interactions between regulatory elements [26,27]. While both STAG proteins contribute to gene regulation, they have distinct roles [26].

*STAG1*-cohesin is more prominent in non-dividing (post-mitotic) cells, where it supports chromatin organization and transcription regulation. It is involved in DNA double-strand break repair, thereby preventing genomic instability. It helps recruit repair proteins and ensures proper homologous recombination, reducing the risk of mutations.

Alongside *STAG2*, *STAG1* ensures cell division by maintaining chromatid cohesion during mitosis and meiosis, ensuring proper chromosome segregation [28,29]. *STAG2*-cohesin is preferentially active in mitotic cells, playing a greater role in active chromosome segregation [27]. Although *STAG2* genetic changes are more commonly linked to cancer, some *STAG1* variants have been implicated in tumorigenesis by affecting chromatin architecture and leading to oncogenic gene misregulation [29,30].

*STAG1* pathogenic variants are associated with a spectrum of neurodevelopmental disorders, collectively named cohesinopathies. To date, approximately 20 cases have been documented, each presenting a unique combination of clinical features [2,3,4,5,6,10]. Comparing our patient’s presentation with these reported cases offers valuable insights into phenotypic variability and potential management strategies.

*STAG1* cohesinopathies commonly describe ID, growth defects, and craniofacial abnormalities [4,5,6], as well as developmental delay and behavioural abnormalities [2,3,4,5,6]. The degree of delay may vary, even among monozygotic twins harboring the same *de novo STAG1* variant [2].

Features such as short stature, failure to thrive, scoliosis, vertebral anomalies, and rib fusion have been documented, particularly in individuals with *STAG2* variants, but are also observed in *STAG1*-related cases [10,31,32].

Common facial characteristics include a wide mouth and deep-set eyes. Additional features, such as synophrys (joined eyebrows), long eyelashes, a broad nasal bridge, anteverted nares, a long philtrum, a thin upper lip, and micrognathia, have been observed, though not consistently across all patients. A subset of patients presents with mild microcephaly [5].

Seizures have been reported in several individuals, suggesting a predisposition to epilepsy within this patient population. Some patients exhibit structural anomalies, including congenital clubfoot and microphthalmia (abnormally small eyes) [10].

While not as pronounced as in other cohesinopathies like Cornelia de Lange Syndrome (CdLS) [8,9,33], subtle limb anomalies, such as fifth finger clinodactyly (curved little finger) and syndactyly (fusion of fingers or toes), have also been noted.

Noticeably, the phenotypic spectrum of *STAG1*-related disorders is broad and can vary significantly among individuals. This heterogeneity may be due to transcriptional dysregulation caused by defects in the cohesin complex rather than by specific genetic alterations [4].

### 3.2. CPP in This Case’s Context

Our patient exhibited CPP, a feature that has not been described in association with *STAG1* variants [17].

CPP is characterized by the early activation of the hypothalamic-pituitary-gonadal (HPG) axis, leading to premature sexual development. The onset of CPP is complex: genetic factors contributing to the condition have been shown to act through loss or gain of function variation [34,35,36] in various genes, as well as through susceptibility [37]; the timing of puberty is often influenced by epigenetic [38] and environmental factors [36]. Most cases remain idiopathic.

Its main feature is the early onset of puberty, occurring before 8 years of age in girls and before 9 years of age in boys. This early onset is caused by the release of gonadotropin-releasing hormones (GnRH) and the stimulation of the pituitary gland to release gonadotropins, which, in turn, stimulate the ovaries or testes to produce sex hormones.

Based on the existing literature, the etiology of CPP involves a complex interplay of genetic, epigenetic, and environmental factors. Loss-of-function mutations in the *MKRN3* and *DLK1* genes are significant contributors to CPP [34]. Gain-of-function mutations in the *KISS1* and *KISS1R* genes, crucial for the regulation of GnRH release, have been linked to CPP [35], as well as variants in genes such as *LIN28B*, *GABRA1*, and *TAC3/TACR3* [36,37]. We have thoroughly examined these genes as part of the genetic testing for our case.

Susceptibility can also play a role. Specific variants in the ERα (rs2234693 and rs9340799) and ERβ (rs1256049) genes have been identified as susceptibility factors for precocious puberty [37]. These are not currently part of the diagnostic process and have not been evaluated.

Epigenetic mechanisms may be involved. The methylation status of *MKRN3* and *DLK1* can influence their expression and, consequently, the timing of puberty [34].

Environmental factors, such as exposure to secondhand smoke and a high BMI, may also contribute [36]. Of note, our patient was consistently overweight, although the initial BMI was only slightly above normal. We cannot evaluate how this, or other endocrine disruptors in her environment, have influenced the onset of CPP.

Could *STAG1,* in fact, be the link between neurodevelopmental aspects and endocrine involvement? Extrapolating from similar disorders, we have seen that abnormalities in reproductive development or even early puberty can be linked to a few cohesinopathies [3,39].

Individuals with CdLS, Kabuki Syndrome, or Charge syndrome have been reported to experience early puberty or other reproductive system abnormalities [3,18,33,40]. *NIPBL*, one of the genes associated with CdLS, has been linked to hormonal dysregulation [12,41]. Recent reported data have found rare *NIPBL* variants in individuals with congenital hypogonadotropic hypogonadism (CHH), a condition characterized by delayed or absent puberty due to inadequate secretion of gonadotropin-releasing hormone (GnRH). These discoveries imply that *NIPBL* variants may hinder the development or function of GnRH neurons, resulting in deficiencies in reproductive hormones. Moreover, *NIPBL* levels have been shown to influence *STAG*-related chromatin dynamics [42]. In terms of mechanisms, we could speculate that the link between CPP occurrence and *STAG1* may be one of the following possibilities:

(1) *STAG1* mutations may disrupt gene regulatory networks involved in the neuroendocrine control of puberty or epigenetic mechanisms, such as histone modification and chromatin remodeling, which are crucial for the timing of GnRH secretion [13,43].
(2) The neurodevelopmental abnormalities seen in *STAG1*-related disorders may indirectly influence hypothalamic function, leading to the early activation of puberty.

If there is indeed such a link, the exact mechanism by which *STAG1* dysfunction may contribute to pubertal acceleration needs to be explored.

### 3.3. Bone Fragility in This Case’s Context

Another particularly striking aspect of this case is the presence of multiple non-traumatic bone fractures, suggesting underlying bone fragility for which no genetic cause could be identified.

Bone fragility is a multifaceted condition that leads to an increased risk of fractures. Congenital metabolic bone disorders are significant contributors [44], but the condition can also occur due to environmental factors, be idiopathic, or be secondary to other conditions [45].

Over 100 different Mendelian-inherited metabolic bone disorders have been identified, affecting more than 80 genes that are involved in bone and mineral metabolism [44]. Environmental and socioeconomic factors also play a role; for instance, exposure to endocrine-disrupting chemicals like pesticides [46]. Osteoporosis is a well-known cause of bone fragility. It can be idiopathic or secondary to other conditions [45]. With such a complex etiology, it is difficult to delineate what the presence of this symptom teaches us about cohesinopathies.

While skeletal abnormalities are not a well-established feature of *STAG1*-related disorders, the cohesin complex is known to influence bone development and homeostasis [7,27]. Other cohesinopathies, such as CdLS and Roberts syndrome, have been associated with skeletal defects [8,9], raising the possibility that *STAG1* dysfunction may similarly impact bone integrity. To speculate, several potential mechanisms could explain this association, as follows:

(1) Defective osteoblast function or connective tissue involvement is due to the cohesin complex regulating genes involved in osteoblast differentiation and bone matrix formation, or to impaired DNA repair mechanisms.
(2) Hormonal influence on bone health: The early activation of the HPG axis in CPP may lead to accelerated bone age. While early estrogen exposure initially increases bone mineralization, it also leads to premature epiphyseal closure, which may contribute to long-term bone fragility.

It may also be that there is a different, yet undiscovered factor altogether, genetic or otherwise, explains the occurrence of this symptom in our patient.

### 3.4. Challenges and Future Directions

These findings in our case report challenge the known phenotypic spectrum of *STAG1*-related disorders by showing an atypical association of CPP and bone fragility with signs and symptoms that may or may not be attributed to *STAG1* cohesinopathy. Case reports are valuable; as we gain a deeper understanding of *STAG1*-related pathophysiology, clinical presentation, and etiology, they may lead to new therapeutic strategies. There are several limitations that should be acknowledged and future directions to consider.

Since *STAG1*-related disorders may evolve over time, a longer follow-up period is necessary to describe their natural history beyond the pediatric age by monitoring the progression of neurodevelopmental, endocrine, and skeletal symptoms. For instance, regular bone mineral density assessments and hormonal evaluations would help determine whether the fractures and CPP are isolated findings or part of a progressive *STAG1*-related phenotype.

Genetic diagnosis is a crucial step. Nonetheless, as our study shows, genetic diagnosis is not always straightforward. The study does not exclude the presence of additional genetic variants that may contribute to the patient’s phenotype. Periodic reinterpretation of the exome data may identify variants that could provide satisfactory answers to genotype-phenotype questions. Whole-genome sequencing (WGS) could help identify other modifying genetic factors. Environmental factors such as nutrition, hormonal influences, or mechanical stress may also contribute to CPP and susceptibility to fractures, making it challenging to determine whether these features are attributed to the identified *STAG1* variant.

Given the rarity of *STAG1* mutations, there is a lack of extensive literature on potential endocrine or skeletal involvement. Are these findings truly part of the disorder’s spectrum? Further studies are required to assess whether these particular features are directly linked to *STAG1* or to other yet unproven genetic or non-genetic causes. Larger case series or cohort studies would be needed to establish a genotype–phenotype correlation, along with, in the case of rare diseases such as this, convincing functional data to unequivocally prove causality. Functional studies, methylation analysis, RNA sequencing, protein expression analysis, or cellular modeling could provide mechanistic insights.

Given the rarity and phenotypic variability of *STAG1*-related disorders, clinical guidelines have not yet been established. However, insights can be drawn from the management of other cohesinopathies, such as CdLS, which share overlapping features [8,9,33,40]. Early intervention is crucial for addressing developmental delays. This includes individualized educational programs and therapies, such as speech, occupational, and physical therapy, to support cognitive and motor development. Regular neuropsychological assessments can help monitor progress and adjust interventions accordingly. Genetic counseling is recommended to discuss the implications of genetic findings, recurrence risks, and family planning. Connecting the family with patient advocacy groups can offer valuable resources and community support.

The variability in clinical presentations among individuals with *STAG1* mutations underscores the necessity for personalized, multidisciplinary management approaches. Furthermore, this case report emphasizes the importance of individualized patient management strategies in complex overlapping phenotypes such as intellectual disability, CPP, and bone fragility, regardless of their common aetiology or lack thereof. In our case, regular neurodevelopmental assessments, vigilant monitoring for endocrine abnormalities such as CPP, and proactive evaluation of bone health, including potential treatment with bisphosphonates, are critical components of comprehensive care for our patient. Coordination among various specialties is therefore vital; this includes regular follow-ups with neurology, endocrinology, orthopaedics, and genetics to monitor and address emerging health concerns.

## 4. Conclusions

This case report associates ID, CPP, and bone fragility. Phenotypes such as the one presented here highlight the difficulty of diagnosing complex clinical phenotypes; genetic testing may only be able to partially uncover the etiology. The current understanding of *STAG1* disease is limited; the association of clinical phenotypes that we show challenges how the clinical spectrum of *STAG1*-related disorders is defined. It is currently unknown whether CPP and bone fragility have common molecular mechanisms with the underlying *STAG1* disease. A deeper understanding of genotype–phenotype associations is needed.

## Data Availability

Data sharing is not applicable to this article.

## Abbreviations

The following abbreviations are used in this manuscript:

ID	Intellectual disability
CPP	Central precocious puberty
DNA	Deoxyribonucleic acid
CdLS	Cornelia de Lange Syndrome
ADHD	Attention-deficit/hyperactivity disorder
LH	Luteinizing hormone
FSH	Follicle-stimulating hormone
MRI	Magnetic resonance imaging
EEG	Electroencephalography
MLPA	Multiplex Ligation-dependent Probe Amplification
NGS	Next-generation sequencing
WES	Whole-exome sequencing
HPG axis	Hypothalamic–pituitary–gonadal axis
GnRH	Gonadotropin-releasing hormone
BMI	Body Mass Index
WGS	Whole-genome sequencing
RNA sequencing	Ribonucleic acid sequencing

## References

[1] Barbero J. (2012). Cohesins, cohesin-regulators: Role in Chromosome Segregation/Repair, Potential in Tumorigenesis. Atlas Genet. Cytogenet. Oncol. Haematol..

[2] Cipriano L., Russo R., Andolfo I., Manno M., Piscopo R., Iolascon A., Piscopo C. (2024). A Novel De Novo STAG1 Variant in Monozygotic Twins with Neurodevelopmental Disorder: New Insights in Clinical Heterogeneity. Genes.

[3] Yuan B., Neira J., Pehlivan D., Santiago-Sim T., Song X., Rosenfeld J., Posey J.E., Patel V., Jin W., Adam M.P. (2019). Clinical exome sequencing reveals locus heterogeneity and phenotypic variability of cohesinopathies. Genet. Med..

[4] Di Muro E., Palumbo P., Benvenuto M., Accadia M., Di Giacomo M.C., Manieri S., Abate R., Tagliente M., Castellana S., Mazza T. (2021). Novel STAG1 Frameshift Mutation in a Patient Affected by a Syndromic Form of Neurodevelopmental Disorder. Genes.

[5] Funato M., Uehara T., Okada Y., Kaneko H., Kosaki K. (2022). Cohesinopathy presenting with microtia, facial palsy, and hearing loss caused by STAG1 pathogenic variant. Congenit. Anom..

[6] Lehalle D., Mosca-Boidron A.L., Begtrup A., Boute-Benejean O., Charles P., Cho M.T., Clarkson A., Devinsky O., Duffourd Y., Duplomb-Jego L. (2017). STAG1 mutations cause a novel cohesinopathy characterised by unspecific syndromic intellectual disability. J. Med. Genet..

[7] Labudina A.A., Meier M., Gimenez G., Tatarakis D., Ketharnathan S., Mackie B., Schilling T.F., Antony J., Horsfield J.A. (2024). Cohesin composition and dosage independently affect early development in zebrafish. Development.

[8] Avagliano L., Parenti I., Grazioli P., Di Fede E., Parodi C., Mariani M., Kaiser F.J., Selicorni A., Gervasini C., Massa V. (2020). Chromatinopathies: A focus on Cornelia de Lange syndrome. Clin. Genet..

[9] Deschamps G.N. (2022). Cornelia de Lange Syndrome. Neonatal Netw..

[10] Bregvadze K., Sukhiashvili A., Lartsuliani M., Melikidze E., Tkemaladze T. (2024). A novel STAG1 variant associated with congenital clubfoot and microphthalmia: A case report. SAGE Open Med. Case Rep..

[11] Canton A.P.M., Seraphim C.E., Montenegro L.R., Krepischi A.C.V., Mendonca B.B., Latronico A.C., Brito V.N. (2024). The genetic etiology is a relevant cause of central precocious puberty. Eur. J. Endocrinol..

[12] Moise-Silverman J., Silverman L.A. (2022). A review of the genetics and epigenetics of central precocious puberty. Front. Endocrinol..

[13] Brito V.N., Canton A.P., Seraphim C.E., Abreu A.P., Macedo D.B., Mendonca B.B., Kaiser U.B., Argente J., Latronico A.C. (2023). The congenital and acquired mechanisms implicated in the etiology of central precocious puberty. Endocr. Rev..

[14] Ward L.M. (2021). Part I: Which child with a chronic disease needs bone health monitoring?. Curr. Osteoporos. Rep..

[15] Charoenngam N., Cevik M.B., Holick M.F. (2020). Diagnosis and management of pediatric metabolic bone diseases associated with skeletal fragility. Curr. Opin. Pediatr..

[16] Richards S., Aziz N., Bale S., Bick D., Das S., Gastier-Foster J., Grody W.W., Hegde M., Lyon E., Spector E. (2015). Standards and guidelines for the interpretation of sequence variants: A joint consensus recommendation of the American College of Medical Genetics and Genomics and the Association for Molecular Pathology. Genet. Med..

[17] Roberts S.A., Kaiser U.B. (2020). GENETICS IN ENDOCRINOLOGY: Genetic etiologies of central precocious puberty and the role of imprinted genes. Eur. J. Endocrinol..

[18] Cheng H., Zhang N., Pati D. (2020). Cohesin subunit RAD21: From biology to disease. Gene.

[19] Zhao B., Lin J., Rong L., Wu S., Deng Z., Fatkhutdinov N., Zundell J., Fukumoto T., Liu Q., Kossenkov A. (2019). ARID1A promotes genomic stability through protecting telomere cohesion. Nat. Commun..

[20] Soldin O.P., Hoffman E.G., Waring M.A., Soldin S.J. (2005). Pediatric reference intervals for FSH, LH, estradiol, T3, free T3, cortisol, and growth hormone on the DPC IMMULITE 1000. Clin. Chim. Acta.

[21] Bangalore Krishna K., Garibaldi L. (2025). Critical appraisal of diagnostic laboratory tests in the evaluation of central precocious puberty. Front. Pediatr..

[22] Caglayan I.S.C., Çelik N., Bolat S. (2024). Evaluation of Serum Adipokine Levels in Girls With Central Precocious Puberty. Clin. Endocrinol..

[23] Çatlı G., Erdem P., Anık A., Abacı A., Böber E. (2015). Clinical and laboratory findings in the differential diagnosis of central precocious puberty and premature thelarche. Turk. Arch. Pediatr./Türk Pediatri Arşivi.

[24] Alghamdi A., Alghamdi A.H. (2023). Precocious puberty: Types, pathogenesis and updated management. Cureus.

[25] Zhao H.-Y., Zhang Y.-R., Zhang R., Li Y.-T., Guo R.-L., Shi W.-S. (2023). Comprehensive analysis of untargeted metabolomics and lipidomics in girls with central precocious puberty. Front. Pediatr..

[26] Ketharnathan S., Labudina A., Horsfield J.A. (2020). Cohesin Components Stag1 and Stag2 Differentially Influence Haematopoietic Mesoderm Development in Zebrafish Embryos. Front. Cell Dev. Biol..

[27] Viny A.D., Bowman R.L., Liu Y., Lavallée V.P., Eisman S.E., Xiao W., Durham B.H., Navitski A., Park J., Braunstein S. (2019). Cohesin Members Stag1 and Stag2 Display Distinct Roles in Chromatin Accessibility and Topological Control of HSC Self-Renewal and Differentiation. Cell Stem Cell.

[28] Arruda N.L., Carico Z.M., Justice M., Liu Y.F., Zhou J., Stefan H.C., Dowen J.M. (2020). Distinct and overlapping roles of STAG1 and STAG2 in cohesin localization and gene expression in embryonic stem cells. Epigenetics Chromatin.

[29] van der Lelij P., Lieb S., Jude J., Wutz G., Santos C.P., Falkenberg K., Schlattl A., Ban J., Schwentner R., Hoffmann T. (2017). Synthetic lethality between the cohesin subunits STAG1 and STAG2 in diverse cancer contexts. Elife.

[30] Saitta C., Rebellato S., Bettini L.R., Giudici G., Panini N., Erba E., Massa V., Auer F., Friedrich U., Hauer J. (2022). Potential role of STAG1 mutations in genetic predisposition to childhood hematological malignancies. Blood Cancer J..

[31] Mullegama S.V., Klein S.D., Signer R.H., Center U.C.G., Vilain E., Martinez-Agosto J.A. (2019). Mutations in STAG2 cause an X-linked cohesinopathy associated with undergrowth, developmental delay, and dysmorphia: Expanding the phenotype in males. Mol. Genet. Genom. Med..

[32] Yingjun X., Wen T., Yujian L., Lingling X., Huimin H., Qun F., Junhong C. (2015). Microduplication of chromosome Xq25 encompassing STAG2 gene in a boy with intellectual disability. Eur. J. Med. Genet..

[33] Della Giustina E., Salviato T., Caramaschi S., Fabbiani L., Reggiani Bonetti L. (2024). Cornelia de Lange Syndrome: Expanding the Neuropathological Spectrum and Clinical Correlations. Fetal Pediatr. Pathol..

[34] Canton A.P.M., Macedo D.B., Abreu A.P., Latronico A.C. (2025). Genetics and Epigenetics of Human Pubertal Timing: The Contribution of Genes Associated With Central Precocious Puberty. J. Endocr. Soc..

[35] Kwon A. (2023). Genetic and epigenetic aspects of the KISS1 and KISS1R genes in pubertal development and central precocious puberty: A review. Precis. Future Med..

[36] Malay J., George B.T., Dube R., Kar S.S., Rangraze I.R. (2024). Exploring the Rise in Precocious Puberty: Interplay of Genetic and Environmental Factors. Preprints.

[37] Gu X., Xiong W., Yang Y., Li H., Xiong C. (2024). A comprehensive meta-analysis to identify susceptibility genetic variants for precocious puberty. Ann. Hum. Genet..

[38] Narusawa H., Ogawa T., Yagasaki H., Nagasaki K., Urakawa T., Saito T., Soneda S., Kinjo S., Sano S., Mamada M. (2024). Comprehensive study on central precocious puberty: Molecular and clinical analyses in 90 patients. J. Clin. Endocrinol. Metab..

[39] Heddar A., Dessen P., Flatters D., Misrahi M. (2019). Novel STAG3 mutations in a Caucasian family with primary ovarian insufficiency. Mol. Genet. Genom..

[40] Seymour H., Feben C., Nevondwe P., Kerr R., Spencer C., Mudau M., Honey E., Lombard Z., Krause A., Carstens N. (2024). Mutation profiling in South African patients with Cornelia de Lange syndrome phenotype. Mol. Genet. Genom. Med..

[41] Liu J., Zhang Z., Bando M., Itoh T., Deardorff M.A., Clark D., Kaur M., Tandy S., Kondoh T., Rappaport E. (2009). Transcriptional dysregulation in NIPBL and cohesin mutant human cells. PLoS Biol..

[42] Alonso-Gil D., Losada A. (2023). NIPBL and cohesin: New take on a classic tale. Trends Cell Biol..

[43] Maione L., Bouvattier C., Kaiser U.B. (2021). Central precocious puberty: Recent advances in understanding the aetiology and in the clinical approach. Clin. Endocrinol..

[44] Marini F., Giusti F., Iantomasi T., Brandi M.L. (2021). Congenital metabolic bone disorders as a cause of bone fragility. Int. J. Mol. Sci..

[45] Testa E.J., Callanan T.C., Evans A.R., Aaron R.K. (2022). Osteoporosis and fragility fractures. Rhode Isl. Med. J..

[46] Singh V., Pal A.K., Biswas D., Ghosh A., Singh B.P. (2020). Evaluation of environmental and socioeconomic factors contributing to fragility fractures in Indians. medRxiv.

